# Detecting Non-Overlapping Signals with Dynamic Programming

**DOI:** 10.3390/e25020250

**Published:** 2023-01-30

**Authors:** Mordechai Roth, Amichai Painsky, Tamir Bendory

**Affiliations:** 1 School of Electrical Engineering, Tel Aviv University, Tel Aviv 6997801, Israel; 2 The Industrial Engineering Department, Tel Aviv University, Tel Aviv 6997801, Israel

**Keywords:** dynamic programming, detection theory, gap statistics

## Abstract

This paper studies the classical problem of detecting the locations of signal occurrences in a one-dimensional noisy measurement. Assuming the signal occurrences do not overlap, we formulate the detection task as a constrained likelihood optimization problem and design a computationally efficient dynamic program that attains its optimal solution. Our proposed framework is scalable, simple to implement, and robust to model uncertainties. We show by extensive numerical experiments that our algorithm accurately estimates the locations in dense and noisy environments, and outperforms alternative methods.

## 1. Introduction

This paper studies the classical problem of detecting signal occurrences in a one-dimensional, noisy measurement. This detection problem appears in various signal-processing applications, such as defects detection [1], radar detection [2], fluorescence imaging [3], ultrasound imaging [4,5], signal synchronization for communication [6], and GPS [7]. In particular, the main motivation of this paper arises from the task of particle picking in single-particle cryo-electron microscopy (cryo-EM): a leading technology to constitute the three-dimensional structure of biological molecules [8,9,10]. The goal of particle picking is to detect the location of particle images in a noisy measurement. This problem is especially challenging since the sought particle images might be densely packed and the signal-to-noise ratio (SNR) is low [11,12,13]; our model can be viewed as a one-dimensional version of this task. In particular, motivated by cryo-EM, we focus on detecting fixed and non-overlapping signals, contaminated by additive Gaussian noise.

Let $y\in R^{N}$ be a measurement of the form

(1) $$y\left[n\right]=\sum_{k=1}^{K}x\left[n-n_{k}\right]+\varepsilon \left[n\right],$$

where $n_{1},\ldots ,n_{K}$ are the unknown locations we aim to estimate, $x\in R^{L}$ is the signal, and $\varepsilon \left[n\right]∼N\left(0,\sigma^{2}\right)$ is i.i.d. Gaussian noise. In Section 2, we first assume that the signal *x*, the noise level $\sigma^{2}$, and the number of signal occurrences *K* are known. Later, in Section 3, we extend the method to account for an unknown number of signal occurrences. In Section 4 we demonstrate numerically that the method is also robust to uncertainties in the signal’s length. We allow the locations of the signal occurrences to be arbitrarily spread in the measurement, with a single restriction: the signal occurrences do not overlap, namely,

(2) $$|n_{i}-n_{j}|\geq L\;\mathrm{for}\;\mathrm{all}\;i\neq j;$$

we refer to this restriction as the separation condition. We also define another separation condition for well-separated signals, where the signals are spaced with a minimum distance of a full signal length from each other, namely,

(3) $$|n_{i}-n_{j}|\geq 2L\;\mathrm{for}\;\mathrm{all}\;i\neq j.$$

Assuming the noise level $\sigma^{2}$, the signal *x*, and *K* are known, maximizing the likelihood function of (1) is equivalent to the least squares problem:

$$\begin{array}{cc} \arg\min_{{\hat{n}}_{1},\ldots ,{\hat{n}}_{K}} & {∥y-\sum_{k=1}^{K}x\left[n-{\hat{n}}_{K}\right]∥}_{2}^{2}. \end{array}$$

Thus, it can be readily seen that maximizing the likelihood function under the separation condition (2) is equivalent to the constrained optimization problem:

(4) $$\begin{array}{cc} \; & \arg\max_{{\hat{n}}_{1},\ldots ,{\hat{n}}_{K}}\sum_{k=1}^{K}\sum_{n=0}^{N-1-L}y\left[n\right]x\left[n-{\hat{n}}_{K}\right]\\ & \;\mathrm{subject}\;\mathrm{to}\;\;|{\hat{n}}_{i}-{\hat{n}}_{j}|\geq L\;\mathrm{for}\;\mathrm{all}\;i\neq j. \end{array}$$

Solving this optimization problem accurately and efficiently is the main focus of this paper.

**Figure 1:**
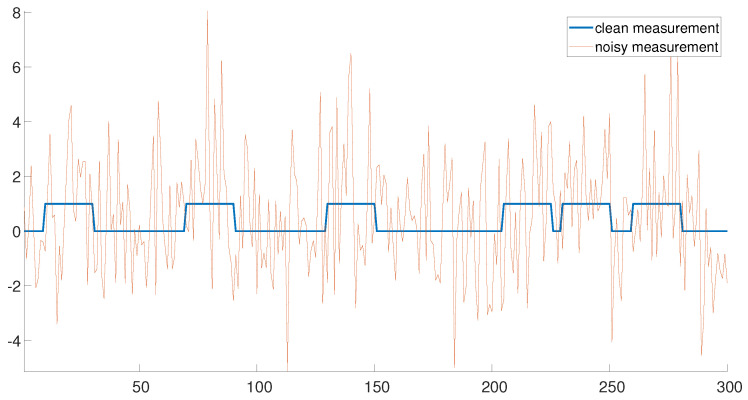
An example of a clean measurement ($\sigma^{2}=0$) and a noisy measurement with $\sigma^{2}=2$.

Figure 1 demonstrates an example of a clean measurement ($\sigma^{2}=0$) and a noisy measurement with $\sigma^{2}=2$. The clean measurement consists of six signal occurrences. Note that the three signal occurrences on the left are well separated. In this regime, the detection problem is rather easy. On the contrary, the three signal occurrences on the right are densely packed, rendering the signal detection problem challenging. Our goal is to estimate the locations of the signal occurrences accurately and efficiently in both regimes.

If the signal occurrences are well separated (the left end of Figure 1), the signal locations may be detected using the following greedy approach. First, the measurement is correlated with the signal *x* (assumed to be known) and the index corresponding to the maximum of the correlation is chosen as the first estimator ${\hat{n}}_{1}$. Next, ${\hat{n}}_{2}$ is chosen as the index corresponding to the maximum of the correlation, where the maximum is taken among all entries which are separated by at least *L* entries from ${\hat{n}}_{1}$. The same strategy is applied consecutively to estimate ${\hat{n}}_{3},\ldots ,{\hat{n}}_{K}$. Hereafter, we refer to this algorithm as the *greedy algorithm*. This algorithm is highly efficient, as the correlations may be executed with only a few FFTs [14]. The greedy approach is a very popular scheme in many real-world applications [15,16]. However, this simple approach fails in cases where the signals are close, as demonstrated in the right end of Figure 1.

The main contribution of this paper is an exact and efficient algorithm to maximize the likelihood function (4). In Section 2, we describe how this maximum is attained by utilizing dynamic programming. Based on the principle of gap statistics, Section 3 extends the scope of our problem and studies the case where the number of signal occurrences *K* is unknown. In Section 4, we conduct comprehensive numerical experiments to study the performance of the proposed dynamic program, its robustness, and compare it to the greedy algorithm. In Section 4.5, we also compare the dynamic program with a convex program that was developed in the context of super-resolution [17,18,19,20]. Finally, we conduct a few experiments on one-dimensional stripes of cryo-EM data (the original data is two-dimensional), indicating that the dynamic program can estimate the locations of densely packed particle images, while the greedy algorithm fails. We conclude the paper in Section 6 by discussing the challenges of extending this framework to two-dimensional data, such as cryo-EM data sets.

## 2. Dynamic Programming for Signal Detection

Dynamic programming is a method for breaking down a problem into simpler sub-problems and solving them recursively [21]. In particular, our proposed dynamic program solution is based on the following procedure. Let g[n,j] be the maximum of (4), for *j* signal occurrences, over indices 1,⋯,n. By definition, g[N,K] is the sought solution of (4). Our proposed dynamic program rule is given by

(5) $$\begin{array}{cc} g[n+1, & j]=\max\left\{g[n,j],g[n-d,j-1]+f[n+1]\right\}, \end{array}$$

where

(6) $$f\left[m\right]=\sum_{i=m-d}^{m+d}y\left[i\right]x\left[i-\left(m-d\right)\right],$$

is the correlation between the measurement *y* at the interval [m−d,m+d] and the signal *x*, while d=⌊L/2⌋ is half the length of the signal. In words, the maximal objective for locating *j* signals over indices 1,…,n+1, is the maximum between the following two options:

1. The best we can achieve for locating *j* signals over indices 1,…,n (namely, the solution of the previous step);
2. The best we can achieve under the constraint that a signal is located at location n+1.

The dynamic program rule introduces a simple *bottom up* routine for finding the maximum of the objective g[N,K]. That is, we define a matrix *g* of dimensions N×K, where each row corresponds to the indices of the measurement and each column is the number of signal occurrences. Then, we iterate over j=1,…,K and i=1,…,N, and fill the entries g[i,j] according to (5). Notice that for every g[i,j], we also store the corresponding estimated signal locations ${\hat{n}}_{1},\ldots ,{\hat{n}}_{j}$. Finally, we return g[N,K], and the corresponding signal locations, as desired. Algorithm 1 summarizes our proposed scheme. Notice that a signal cannot be located near the staring and end indices, namely, at i<L and i>N−L. This means that g[i,j]=0 for all i<L and g[i,j]=g[i−1,j] for all i>N−L. We exclude these cases from the description of Algorithm 1 for simplicity of presentation.
**Algorithm 1:** Signal detection using dynamic programming. **Input:**
$y\in R^{N}$, $x\in R^{L}$, and the number of signal occurrences *K*   1:**for**k=1 to *K* **do**   2:   **for** i=1 to N **do**   3:     compute g[i,k] according to (5)   4:   **end for**   5:**end for**   6:**return**g[N,K] and estimates of the locations of the signal occurrences ${\hat{n}}_{1},\ldots {\hat{n}}_{K}$

The computational complexity of our algorithm is *O*(N·max{K,logN}), as follows. Computing the cross correlation between *y* and *x* costs *O*(NlogN) operations using the FFT algorithm. Given the cross correlation values, every iteration of Algorithm 1 is of *O*(1). Overall, we have *O*(NK) iterations, and thus the computational complexity of the entire proposed scheme is *O*(N·max{K,logN}).

In Section 4 and Section 5, we compare Algorithm 1 against the greedy algorithm described in Section 1. This algorithm chooses the peaks of the cross correlation between the signal and the measurement, while forcing a separation. Thus, its computational complexity is *O*(NlogN+K). For small *K*, the complexities of both algorithms match. Algorithm 2 summarizes this method.
**Algorithm 2:** Signal detection using the greedy approach. **Input:** $y\in R^{N}$, $x\in R^{L}$, and the number of signal occurrences *K*   1:compute *f* according to (6)   2:**for**i=1 to *K* **do**   3:   ${\hat{n}}_{i}=\;\arg\max\;f$    subject to    $|{\hat{n}}_{i}-{\hat{n}}_{j}|\geq L$ for all j<i   4:   $\gamma_{K}\left[i\right]=\;\max f\;$    subject to    $|{\hat{n}}_{i}-{\hat{n}}_{j}|\geq L$ for all j<i   5:**end for**   6:$\gamma_{K}=\sum_{i=1}^{K}\gamma_{K}\left[i\right]$   7:**return** Estimates of the locations of the signal occurrences ${\hat{n}}_{1},\ldots {\hat{n}}_{K}$, and $\gamma_{K}$

We mention in passing that our problem shares some similarities with the change point detection problem—a well-studied problem in statistics. One popular solution to change point detection is based on dynamic programming [22,23]. Yet, this algorithm is significantly different from the dynamic program in Algorithm 1.

## 3. Estimating the Number of Signal Occurrences Using the Gap Statistics Principle

In many real-world applications, the number of signal occurrences *K* is unknown. This problem is of special interest, as both the greedy algorithm and our proposed scheme assume knowledge of *K*. The classical approach for finding *K* is based on finding a “knee” behavior (also referred to as the *elbow method*). This heuristic suggests solving (4) for different values of *K*, and returning the value that introduces the steepest decrease in the objective value. This approach is perhaps the most popular framework in many related applications, such as clustering  [24], regularization  [25] and others. It was extensively studied and improved over the years, see for example [26,27,28].

In our work, we suggest using the principle of gap statistics: a statistically driven modification of the knee approach, which was first introduced in [24] in the context of estimating the optimal number of clusters in a data set. In their work, Tibshirani et al. [24] showed that in standard clustering, the error measure monotonically decreases as the number of clusters increases; however, from some value of *K* onward, the decrease flattens markedly. This *K* is usually referred to as the “knee” of the plot, and is believed to indicate the optimal number of clusters in the data set.

The gap statistic provides a statistical procedure to formulate the detection of this knee. The key idea of this approach is to standardize the curve of the objective value by comparing it with its expectation under an appropriate null reference. Formally, Tibshirani et al. defined the gap statistic as

$${\mathrm{Gap}}_{N}\left(K\right)=E_{N}^{*}w\left(K\right)-w\left(K\right),$$

where w(K) is the objective value over *K* clusters, and $E_{N}^{*}$ denotes the expectation under a sample of size *N* from a “null” reference distribution. By “null”, we mean a clustering performed on noise. The estimate of the number of classes, denoted by $\hat{K}$, is the value that maximizes ${\mathrm{Gap}}_{N}\left(K\right)$. Intuitively, $\hat{K}$ implies the “strongest” evidence against the null. The gap statistic was extensively studied and applied to many applications [29,30,31,32,33,34].

In this work, we take a similar approach, and suggest estimating the number of signal occurrences based on the gap statistics principle. First, we observe that (4) is monotonically increasing in *K*, where the steepest decrease is expected at the vicinity of the true value of *K*. For a given measurement *y* and a range of values of *K*, we apply Algorithm 1 and find the maximal objective value g(N,K). We note that no additional computations are required at this stage since the dynamic program already computes g(N,j) for j=1,…,K. To apply the gap statistics procedure, we also need to evaluate $E_{N}^{*}g\left(N,K\right)$ for every *K*, that is, the expected objective under the null. Here, we define the null as the case where no signal is embedded in the measurement. Therefore, to approximate $E_{N}^{*}g\left(N,K\right)$ we simply permute the vector *yP* times, drawn i.i.d. from a uniform distribution over all possible permutations, and apply Algorithm 1 on the permuted measurements, ${\tilde{y}}_{1},\ldots ,{\tilde{y}}_{P}$. Notice that by permuting the indices of *y*, we break the embedded signals (if such exist) and attain a vector with K=0 (henceforth, the null). Letting $g_{i}\left(N,K\right)$ be the value of (5) for the permuted measurement ${\tilde{y}}_{i}$, we have $E_{N}^{*}g\left(N,K\right)\approx \frac{1}{P}\sum_{i=1}^{P}g_{i}\left(N,K\right)$ for large enough *P*. Therefore, we approximate the statistical gap by

(7) $${\mathrm{Gap}}_{N}\left(K\right)\approx \frac{1}{P}\sum_{i=1}^{P}g_{i}\left(N,K\right)-g\left(N,K\right),$$

for every *K*, and return the value of *K* which maximizes the gap (similarly to [24]). We summarize our approach in Algorithm 3. Algorithm 4 shows the analog of the greedy algorithm, Algorithm 2, in the case where the number of signal occurrences is unknown and estimated using the gap statistic method.
**Algorithm 3:** Signal detection using dynamic programming with an unknown number of signal occurrences. **Input:**$y\in R^{N}$, $x\in R^{L}$, $K_{\max}$   1:**for**K=1 to $K_{\max}$ **do**   2:   Compute g(N,K) with respect to *y* using Algorithm 1   3:   **for** i=1 to *P* **do**   4:     Compute $g_{i}\left(N,K\right)$ with respect to $y_{i}$ (a permutation of *y*) using Algorithm 1   5:   **end for**   6:   Compute ${\mathrm{Gap}}_{N}\left(K\right)=\frac{1}{P}\sum_{i=1}^{P}g_{i}\left(N,k\right)-g\left(N,k\right)$   7:**end for**   8:Compute $\hat{K}=\arg\max\;{\mathrm{Gap}}_{N}\left(K\right)$   9:**return** $\hat{K}$ (an estimate of the number of signal occurrence in the measurement), $g(N,\hat{K})$, and estimates of the locations of the signal occurrences ${\hat{n}}_{1},\ldots ,{\hat{n}}_{\hat{K}}$

**Algorithm 4:** Signal detection using the greedy approach with an unknown number of signal occurrences.
 **Input:** $y\in R^{N}$, $x\in R^{L}$, $K_{\max}$   1:**for**K=1 to $K_{\max}$ **do**   2:   Evaluate $\gamma_{K}$ with respect to *y* using Algorithm 2   3:   **for** i=1 to *P* **do**   4:     Compute ${\tilde{\gamma }}_{K,i}$ with respect to ${\tilde{y}}_{i}$ (a permutation of *y*) using Algorithm 2   5:   **end for**   6:   Compute ${\mathrm{Gap}}_{N}\left(K\right)=\frac{1}{P}\sum_{i=1}^{P}{\tilde{\gamma }}_{K,i}-\gamma_{K}$   7:
**end for**
   8:**return**$\hat{K}$ (an estimate of the number of signal occurrence in the measurement), and estimates of the locations of the signal occurrences ${\hat{n}}_{1},\ldots ,{\hat{n}}_{\hat{K}}$


## 4. Numerical Experiments

In this section, we compare the proposed dynamic program with alternative methods. We use the $F_{1}$-score to evaluate the performance of the studied methods [35,36,37]. It is formally defined as

(8) $$F_{1}=2\times \frac{\mathrm{precision}\times \mathrm{TPR}}{\mathrm{precision}+\mathrm{TPR}},$$

where *precision* is the ratio of the true positives (correct detections) over all detections, while TPR is the true positive rate, the ratio of true positives over all signal occurrences. In addition, we also report the *recall*, the ratio of true positives over all positives, for completion. In practice, we cannot expect an exact detection of a signal location. Therefore, we follow [38] and declare a true detection if $|{\hat{n}}_{k}-n_{k}|<L/2$. That is, we say that a signal is correctly detected if its estimated location is within L/2 entries from the true location. This convention is quite popular in relevant signal detection literature [38,39]. Further, it is well motivated by our cryo-EM application. Specifically, in cryo-EM particle picking, the displacements are not a big issue since the images are later aligned as part of the refinement algorithm [9].

We begin with synthetic experiments. We generate a measurement *y* as follows. First, we fix the measurement length *N*, the number of signal occurrences *K*, and signal length *L*. Then, we place the first signal at a random location. Next, we draw a new location; if the new location is eligible, then we place it, and if not, we draw a new location. We repeat this process until we place all *K* signals. By eligible location, we mean that the left-most point of the new signal is separated by at least *L* entries from the left-most point of all previous signals for an *arbitrary-spaced measurement* (2) (so the signal occurrences do not overlap) and 2L for a *well-separated measurement* (3). Finally, we add independent and identically distributed white Gaussian noise with zero mean and variance $\sigma^{2}$. The code to reproduce all experiments is publicly available at https://github.com/MordechaiRoth1/Signal-detection-with-dynamic-programming (accessed on 25 January 2023).

### 4.1. Performance for a Known Number of Signal Occurrences

**Figure 2:**
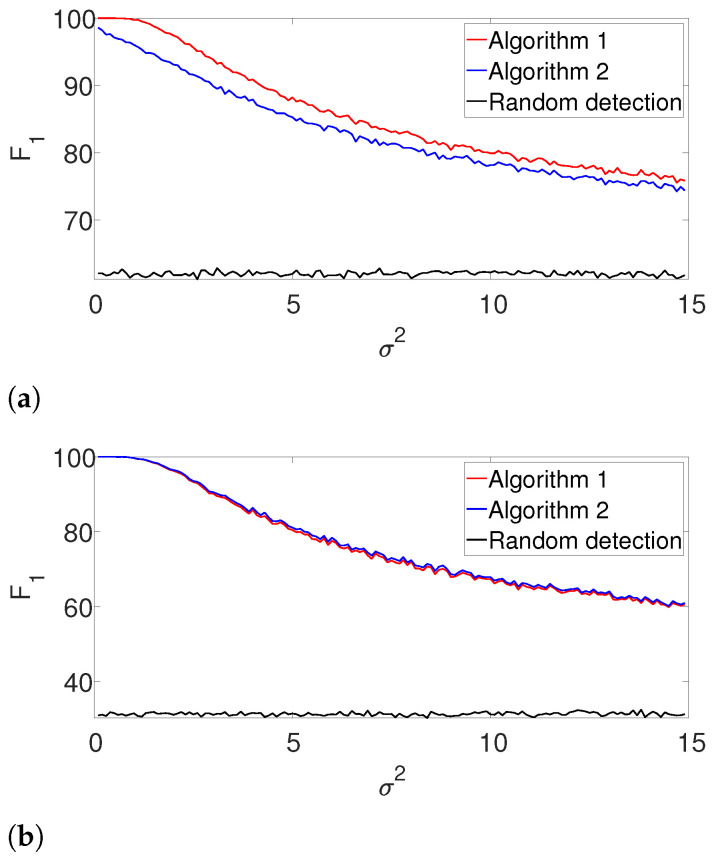
$F_{1}$-score for Algorithms 1 and 2 for the arbitrarily spaced and well-separated setups, assuming the signal’s shape and the number of occurrences are known (**a**) Arbitrarily spaced setup. (**b**) Well-separated setup.

First, we compare the the performance of our dynamic programming scheme (Algorithm 1) with the greedy approach (Algorithm 2) in the ideal case, where the signal’s shape and the number of signal occurrences are known. We use a rectangular signal of length L=30, where all of its entries are equal to one. We place them in a measurement of length N=300, as described above. For the well-separated setup, we set K=3 signal occurrences that satisfy the separation condition (3) and for the arbitrarily spaced case, we use K=6 signal occurrences that only satisfy the *L*-separation condition (2). For each noise level $\sigma^{2}$, we conduct 3000 trials, each with a fresh measurement. As a baseline, we further compute the $F_{1}$-score of a random detection process, where *K* locations which satisfy (2) are chosen at random. Figure 2 presents the results. First, it is evident that the performance of the algorithms are comparable for the well separated case in Figure 2b. However, we observe that the dynamic program outperforms the greedy algorithm in cases where the signals are dense as in Figure 2a. The complementary recall charts are quite similar to the $F_{1}$ scores and are provided in the Appendix A.

### 4.2. Performance for an Unknown Number of Signal Occurrences

**Figure 3:**
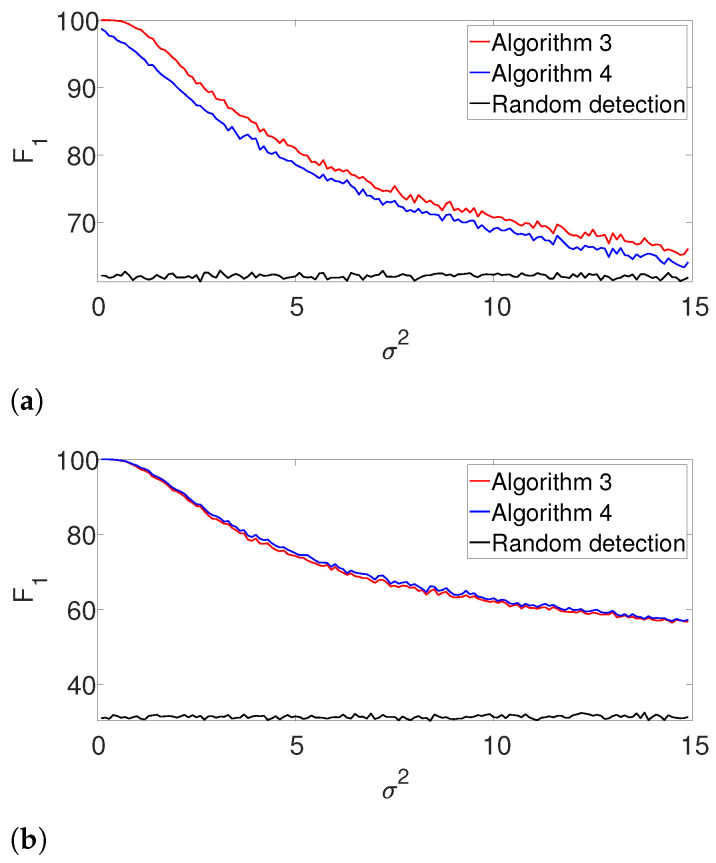
$F_{1}$-score for Algorithm 3 and for Algorithm 4 for the arbitrarily spaced and well-separated setups, assuming the signal’s shape is known but the number of signal occurrences *K* is unknown. (**a**) Arbitrarily spaced setup. (**b**) well separated setup.

Next, we repeat the previous experiment while dropping the known *K* assumption (yet, the signal’s shape is still assumed to be known). In this case, we apply the gap statistic principle to evaluate the number of signal occurrences, while estimating their locations as described in Algorithms 3 and 4. The results are presented in Figure 3. As in the previous example, the performance of both algorithms is comparable for the well-separated case (Figure 3b), while the dynamic program is clearly superior in the arbitrarily spaced (henceforth, dense) setup (Figure 3a). As expected, the performance of the algorithms deteriorates compared to Figure 2. Once again, we report the recall in the Appendix A, as it demonstrates quite a similar behavior.

**Figure 4:**
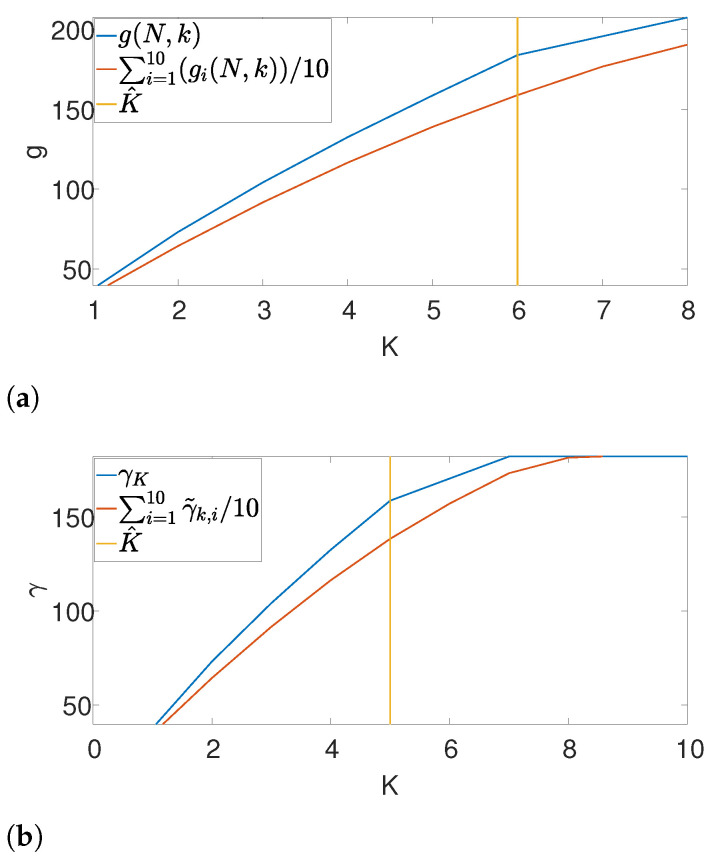
An illustration of the gap statistic principle. The blue curve is the measured objective, while the red curve corresponds to the approximated null. The yellow line is the maximal gap between the two and, henceforth the estimated *K*. (**a**) Gap found by Algorithm 3. (**b**) Gap found by Algorithm 4.

Further, we illustrate our proposed gap statistic scheme in Figure 4. Here, we set the (unknown) number of occurrences as K=6. The blue curve corresponds to the objective value, while the red curve is the approximated null (see (7)). The yellow line corresponds to the maximum gap between the two curves, which is the estimated *K*. As we can see, the gap statistic demonstrates a relatively accurate estimate of the true number of signal occurrences in both algorithms.

### 4.3. Performance with Unknown Signal Length

**Figure 5:**
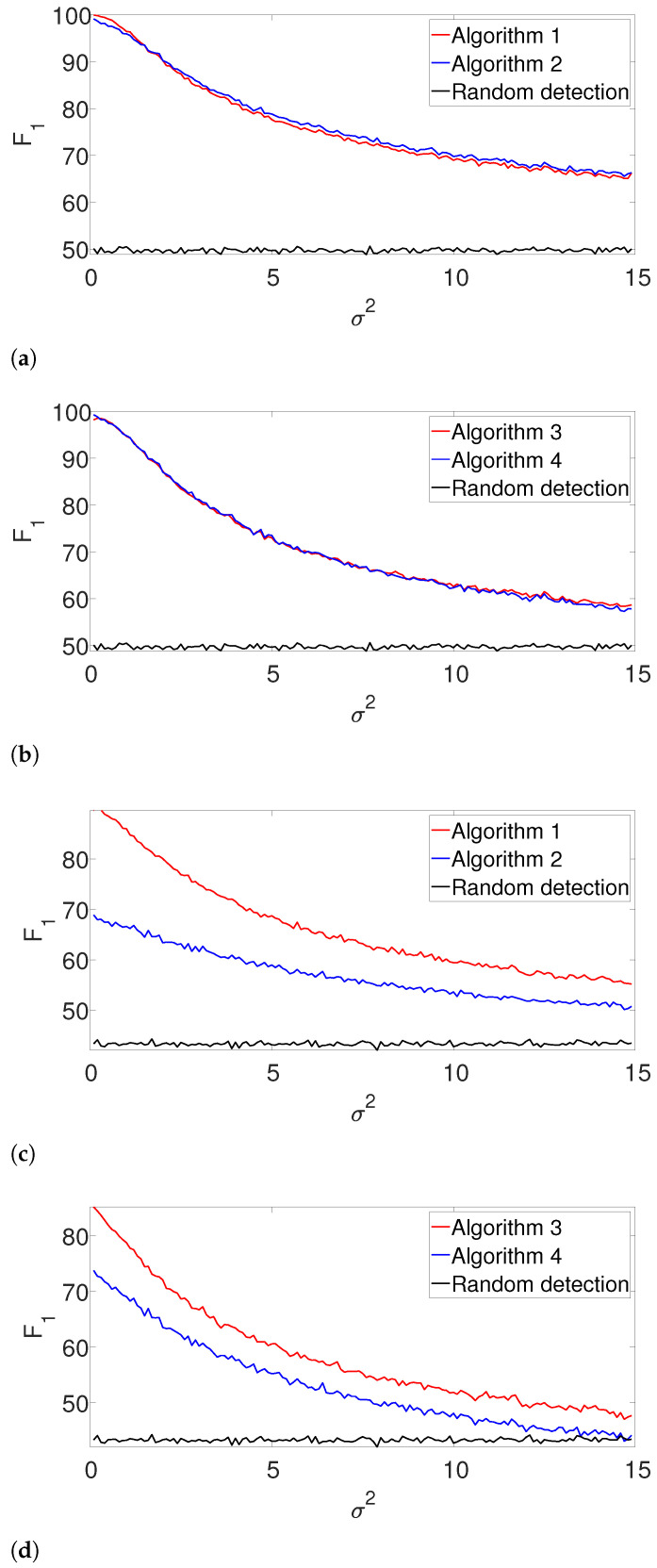
$F_{1}$-score for the arbitrarily spaced model, where the signal length is unknown. Here, $\hat{L}$ denotes the assumed length of the signal. (**a**) $\hat{L}/L=0.8$, known *K*. (**b**) $\hat{L}/L=0.8$, unknown *K*. (**c**) $\hat{L}/L=1.3$, known *K*. (**d**) $\hat{L}/L=1.3$, unknown *K*.

**Figure 6:**
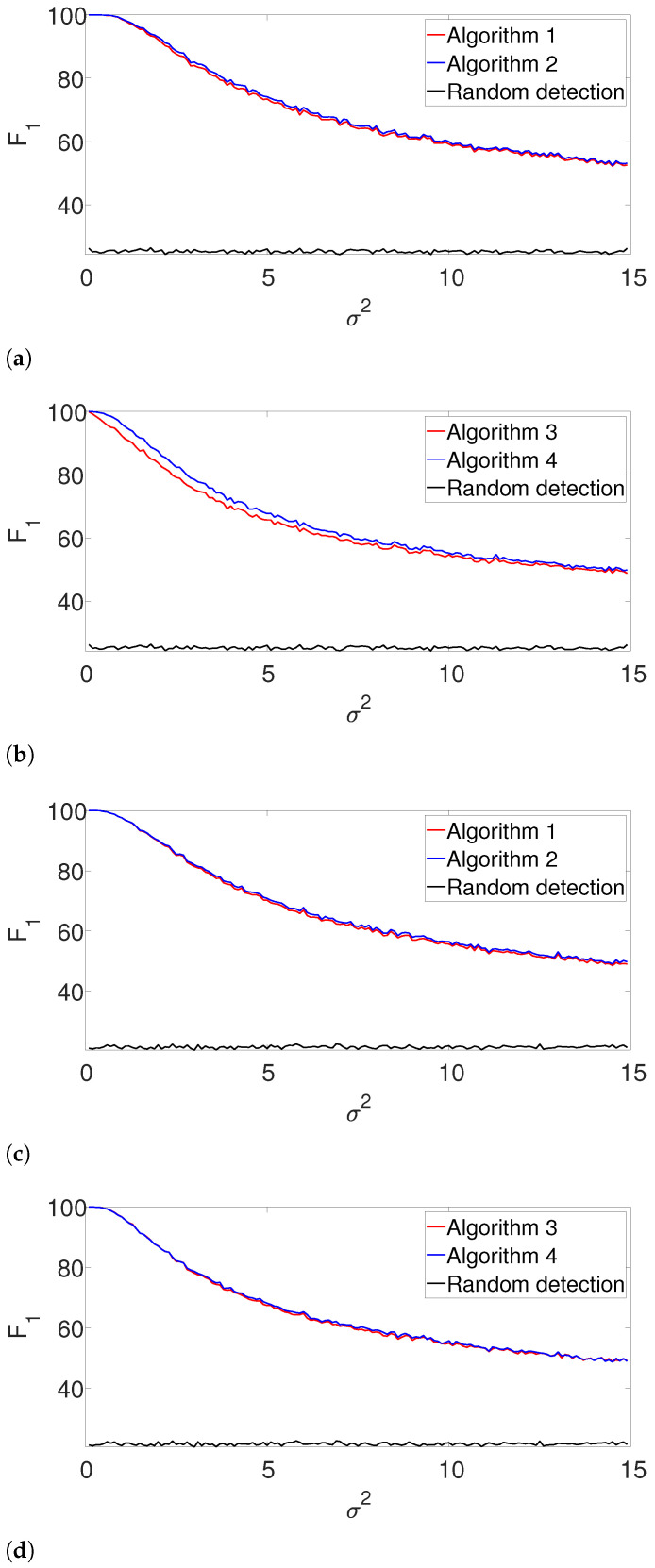
$F_{1}$-score for the well separated model where the signal length is unknown. (**a**) $\hat{L}/L=0.8$, known *K*. (**b**) $\hat{L}/L=0.8$, unknown *K*. (**c**) $\hat{L}/L=1.3$, known *K*. (**d**) $\hat{L}/L=1.3$, unknown *K*.

Further, we study the robustness of our proposed scheme, as we focus on the case where the length of the signal *L* is not precisely known. Let $\hat{L}$ denote the approximated signal length. We examine two cases: $\hat{L}/L=0.8$ (the true signal’s length is greater than its approximation) and $\hat{L}/L=1.3$. We study the performance of our suggested framework in cases where *K* is either known or unknown. The $F_{1}$ results are presented in Figure 5 and Figure 6 for the arbitrarily spaced and well-separated cases, respectively. The complementary recall charts are again reported in the Appendix A.

In the arbitrarily spaced setup, we observe a similar behavior for $\hat{L}/L=0.8$ (Figure 5a,b), while our proposed method outperforms the greedy algorithm for $\hat{L}/L=1.3$ (Figure 5c,d). The reason for this phenomenon can be explained as follows. When $\hat{L}/L=1.3$, the true signal is shorter than assumed. Thus, the greedy algorithm declares close signals as a single realization. For $\hat{L}/L=0.8$, both algorithms perform quite similarly, as our proposed algorithm does not impose a strong enough separation constraint.

Figure 6 shows the $F_{1}$-score for the well-separated setup. Here, the performance of the greedy algorithm is comparable to the dynamic program in all the examined setups. This behavior is not surprising. In the well-separated regime, the separation constraint is less effective, and both algorithms perform quite similarly, regardless of the accuracy of $\hat{L}$.

### 4.4. Performance as a Function of the Measurement Length

**Figure 7:**
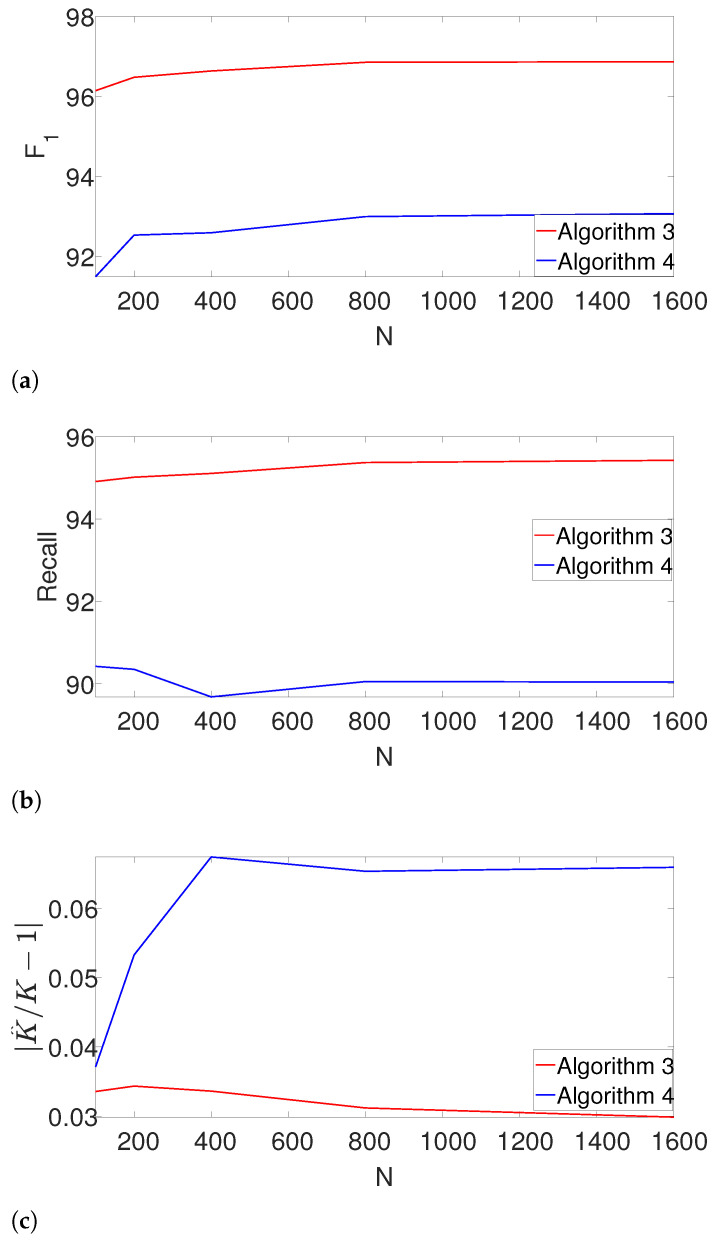
The effect of the measurement length *N* on the estimation accuracy. (**a**) $F_{1}$-score as a function of *N*. (**b**) Recall as a function of *N*. (**c**) The error in estimating the number of signal occurrences $\hat{K}$, $|\hat{K}/K-1|$, as a function of *N*.

Next, we study the performance of the dynamic program as the length of the measurement *N* increases. Here, we set L=20, and fix the density of the signals, so that KL/N=0.6. We further assume that *K* is unknown. In addition to the $F_{1}$-score and the recall, we also measure the accuracy of estimating *K* using the measure $|\hat{K}/K-1|$. The results are presented in Figure 7.

Evidently, Algorithm 3 outperforms Algorithm 4 in terms of $F_{1}$-score, recall and the error of estimating *K*. Note that our proposed scheme is not only robust to the number of signal occurrences as *N* grows, but it also slightly improves.

### 4.5. Comparison with a Convex Optimization Approach

An additional approach to detect signal occurrences is using a convex optimization framework, which was originally developed in the context of super-resolution [17,18]. Here, the underlying idea is to denoise the measurement using a convex program, and then apply a detection algorithm to the denoised measurement.

Here, we describe the noiseless measurement by a matrix-vector multiplication z=Gs, where the *i*-th row of the circulant matrix $G\in R^{N\times N}$ is *x*, padded with zeros and shifted by *i* entries, and $s\in {\left[0,1\right]}^{N}$ is a binary signal containing ones at the left-most entry of the signal occurrences and zeros otherwise. The measurement is given by y=z+ε, where ε is i.i.d. white Gaussian noise with zero mean and variance of $\sigma^{2}$. Consequently, the detection problem is to estimate the binary, sparse vector *s* from the measurement *y*.

Following [18], we suggest estimating *s* by minimizing its $\ell_{1}$ norm subject to the constraint y≈Gs. In addition, we relax the binary constraint to a “box constraint,” resulting in the following convex program:

(9) $$\begin{array}{cc} \; & \min_{s\in R^{N}}{\left||s|\right|}_{1}\;\mathrm{subject}\;\mathrm{to}\;||y-{Gs||}_{2}^{2}\leq \delta \\ \; & \;0\leq s[i]\leq 1,\;i=0,\ldots ,N-1. \end{array}$$

We set $\delta =1.2N\sigma^{2}$.

We solve the convex program (9) using CVX [40], resulting in a denoised measurement. Then, similarly to the procedure of Algorithm 2, we chose to *K* greatest peaks, while enforcing a separation of *L* entries.

**Figure 8:**
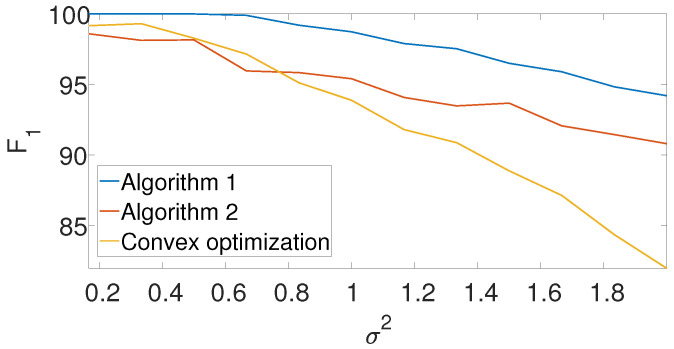
$F_{1}$-score for the arbitrarily spaced model as a function of the noise level for Algorithm 1, Algorithm 2, and the convex program (9).

Figure 8 compares the $F_{1}$-score of the convex program with the dynamic program (Algorithm 1) and the greedy algorithm (Algorithm 2) for different noise levels. The recall is again left for the Appendix A. We set L=15, N=75, and K=3 in the arbitrarily spaced setup. The dimension of the problem is relatively low because of the high computational burden of the convex approach. Evidently, both Algorithms 1 and 2 outperform the convex approach.

## 5. Cryo-EM Numerical Experiment

In the cryo-EM experiment, biological macromolecules suspended in a liquid solution are rapidly frozen into a thin ice layer. An electron beam then passes through the sample, producing a 2D tomographic projection, called a micrograph. The first step in the algorithmic pipeline is detecting the projection images in the micrograph; this process is called particle picking [11,12,13]. Particle picking is particularly challenging since the SNR of cryo-EM is rather low due to the absence of contrast enhancement agents and the low doses of electrons. The detected projection images are later used to reconstruct the 3D structure of the sought molecule [9,10]. The problem studied in this paper may be viewed as a 1D version of the cryo-EM particle-picking process.

**Figure 9:**
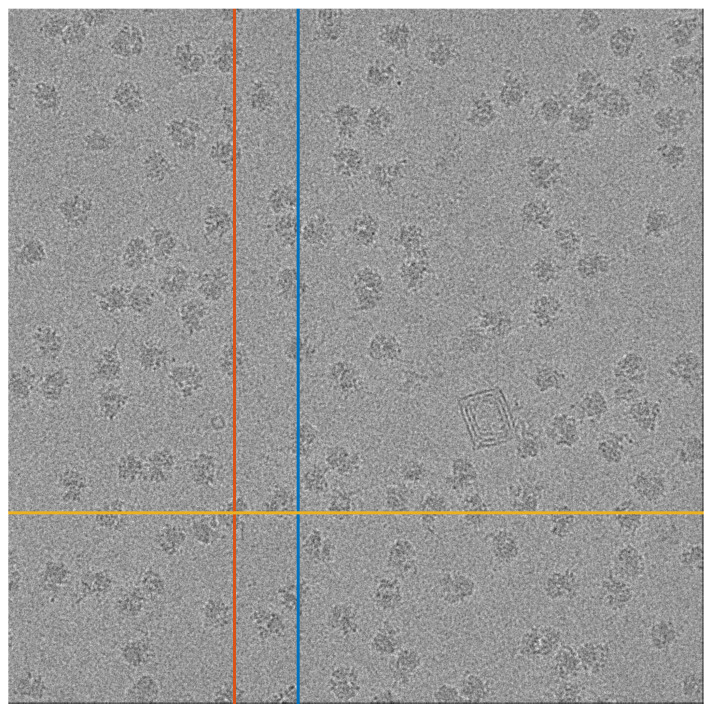
A micrograph from the EMPIAR 10028 data set. The three marked lines are used as inputs for Algorithms 3 and 4. The red and blue lines are columns 1324 and 1697, respectively, and the yellow line is row 2952.

To test our approach, we used a micrograph that contains tomographic projections of the Plasmodium Falciparum 80S ribosome [41]. This data set is publicly available at the EMPIAR repository [42] as EMPIAR 10028. The micrograph is presented in Figure 9. We arbitrarily chose 1D stripes (columns or rows) of the micrograph, on which we can apply our 1D detection algorithm. We note that the particle projections along the 1D stripes are not identical, which is a more complicated regime than the one considered in Section 4.

**Figure 10:**
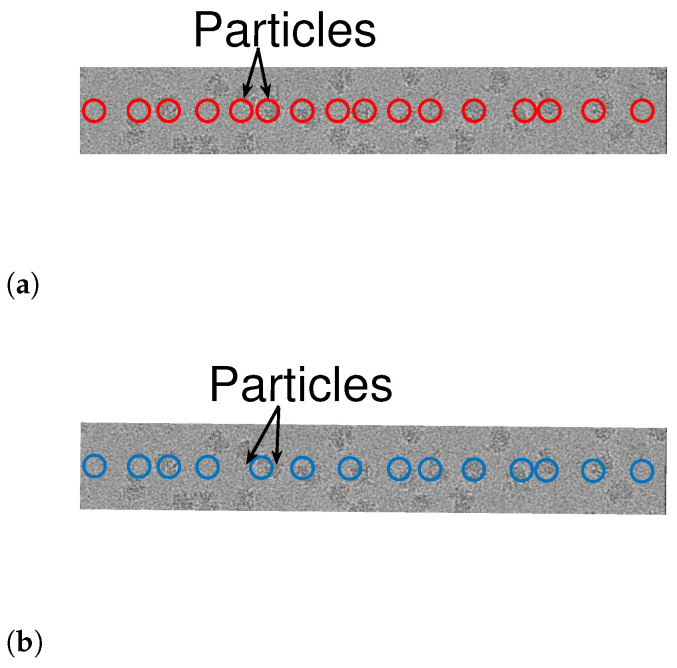
Detection using Algorithms 1 and 2 for row 2952 (yellow line in Figure 9). The arrows point to two particle projections, which are detected using Algorithm 3 but not by Algorithm 4. (**a**) Algorithm 3. (**b**) Algorithm 4.

**Figure 11:**
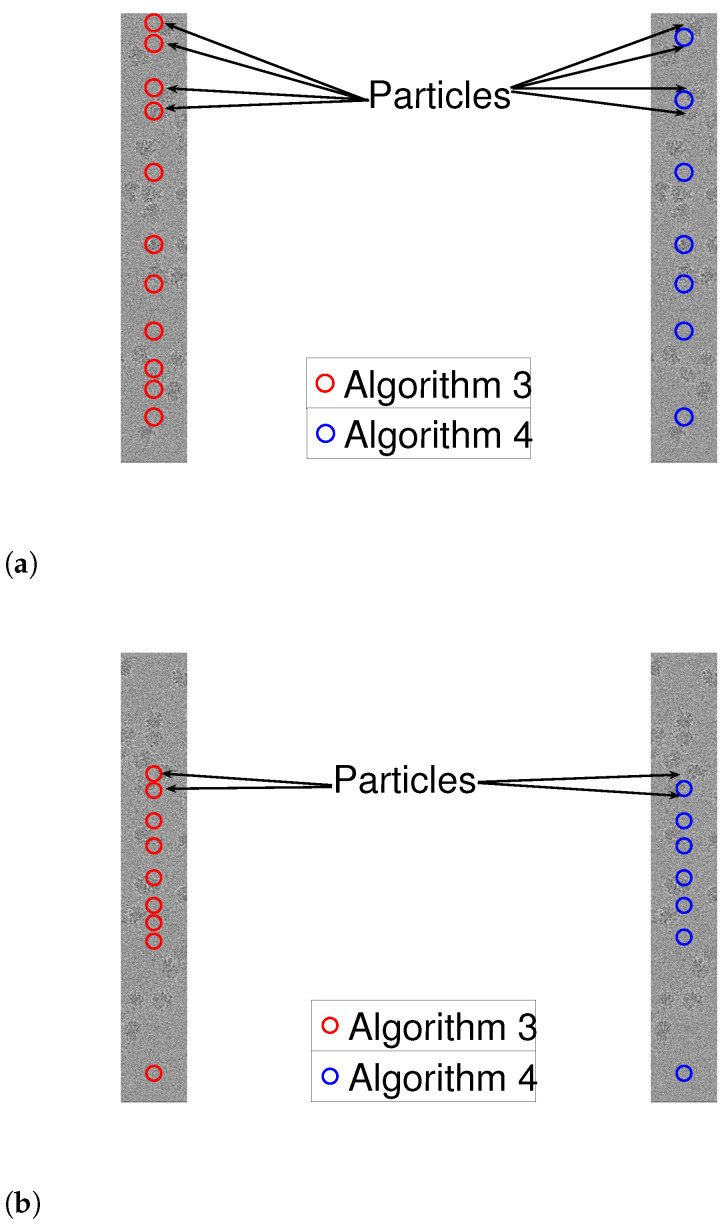
Detection using Algorithms 3 and 4. The arrows point to particle projections, which are detected by Algorithm 3, while Algorithms 4 fails. (**a**) Column 1324 (red line in Figure 9). (**b**) Column 1697 (blue line in Figure 9).

**Figure 12:**
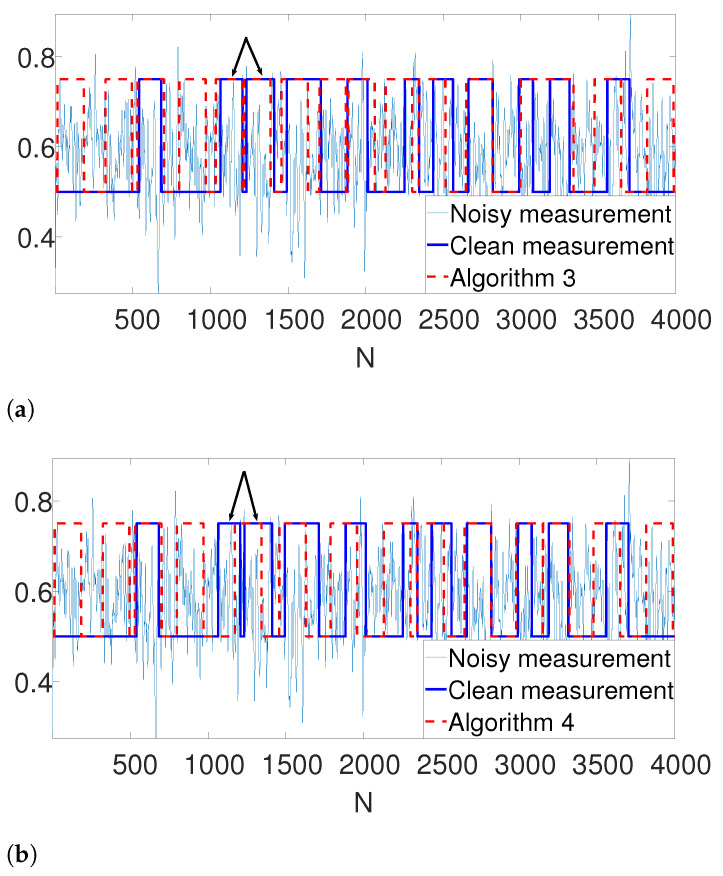
Detection using Algorithms 1 and 2 for row 2952 (yellow line in Figure 9). The arrows point to two particle projections, which are detected by Algorithm 3 and not by Algorithm 4. (**a**) Algorithm 3. (**b**) Algorithm 4.

As a prepossessing step, we whiten the noise, a standard step in many cryo-EM algorithmic pipelines. This is done in the following manner. First, we manually find a region in the measurement with no signal. Using this “noise-only” data, we approximate the power spectral density of the noise. Then, we multiple the entire measurement by the inverse of the approximated power spectrum. We are now ready to apply Algorithms 3 and 4 to 1D measurements, after whitening. We assumed that the shape of the signal is a square pulse whose length is chosen manually. To evaluate the results, we manually tagged the true locations of the particles (namely, signal occurrences). Figure 10 and Figure 11 illustrate the results. While both algorithms are fairly similar in the more sparse environments, the dynamic program approach succeeds in identifying densely packed particles (highlighted with arrows), while the greedy method fails. This indicates that extension of our scheme to 2D images may be helpful to locating densely packed particle images in cryo-EM data sets. In addition, we illustrate 1D projections of our results in Figure 12. As we can see, our proposed scheme successfully detects the particles, while the greedy algorithm demonstrates inferior results.

## 6. Discussion

This papers introduces a novel scheme for signal detection based on a dynamic program that maximizes a constrained likelihood function. We apply the gap statistic principle to estimate the number of signal occurrences, and provide an end-to-end solution to the problem. We demonstrate our proposed method in a series of experiments. Our suggested scheme demonstrates improved performance over popular alternatives in dense environments, while attaining similar results in sparse regimes. This makes it a robust approach in many practical setups.

Our work is motivated by the cryo-EM technology. Typically, particle pickers are based on cross correlating the micrograph with different templates. This approach performs well in cases where the particles are well separated but fails in dense regimes. We show that by imposing a separation constraint, we improve upon currently known schemes in the 1D regime. This motivates our future work, generalizing our results to 2D images, and provides an efficient solution to the cryo-EM particle picking problem.

## Figures and Tables

**Figure A1:**
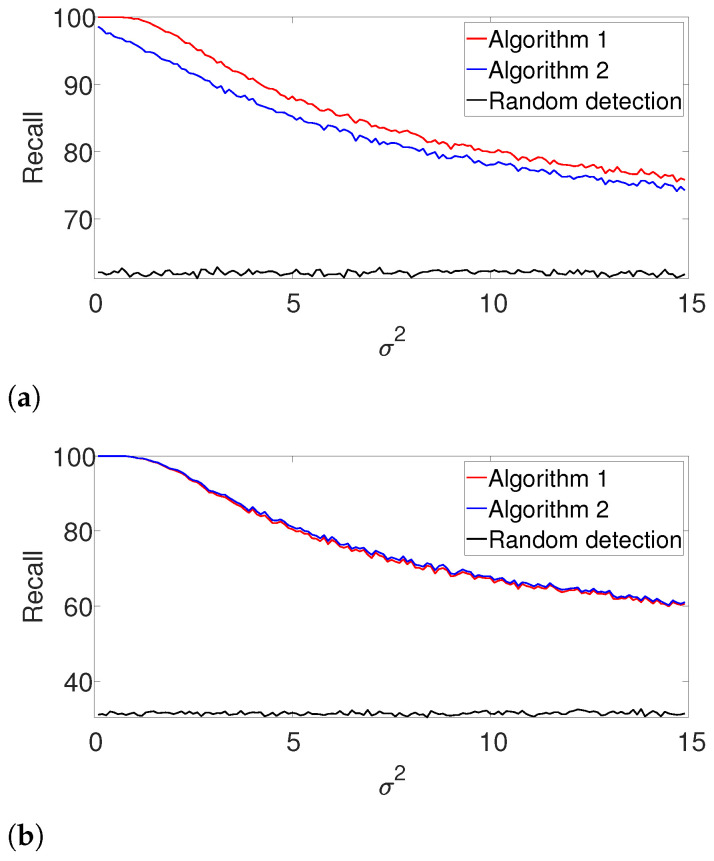
Recall for Algorithms 1 and 2 for the arbitrarily spaced and well-separated setups, assuming the signal’s shape and the number of occurrences are known. (**a**) Arbitrarily spaced setup. (**b**) Well-separated setup.

**Figure A2:**
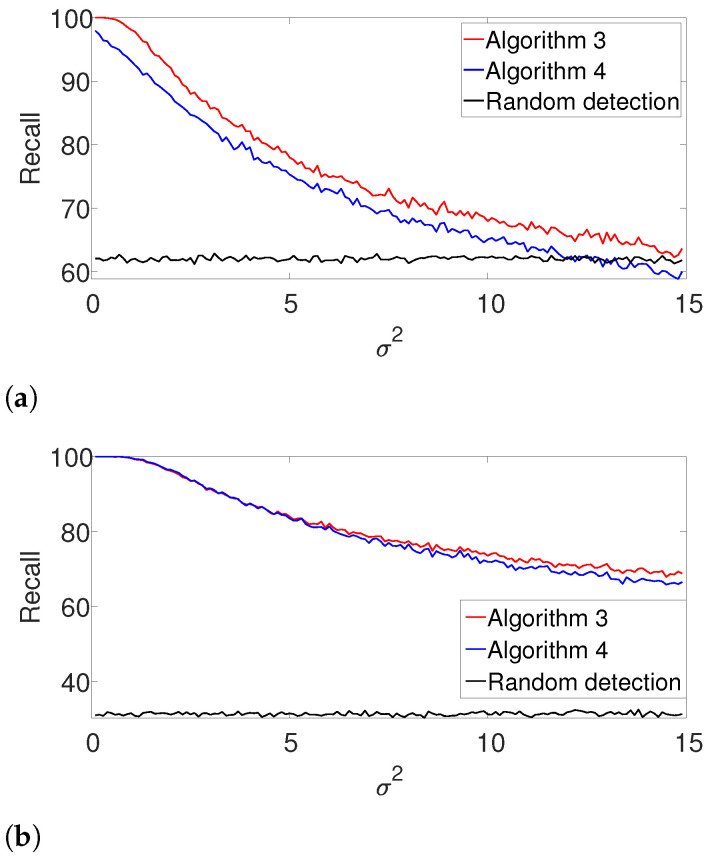
Recall for Algorithm 3 and for Algorithm 4 for the arbitrarily spaced and well-separated setups, assuming the signal’s shape is known but the number of signal occurrences *K* is unknown. (**a**) Arbitrarily spaced setup. (**b**) Well-separated setup.

**Figure A3:**
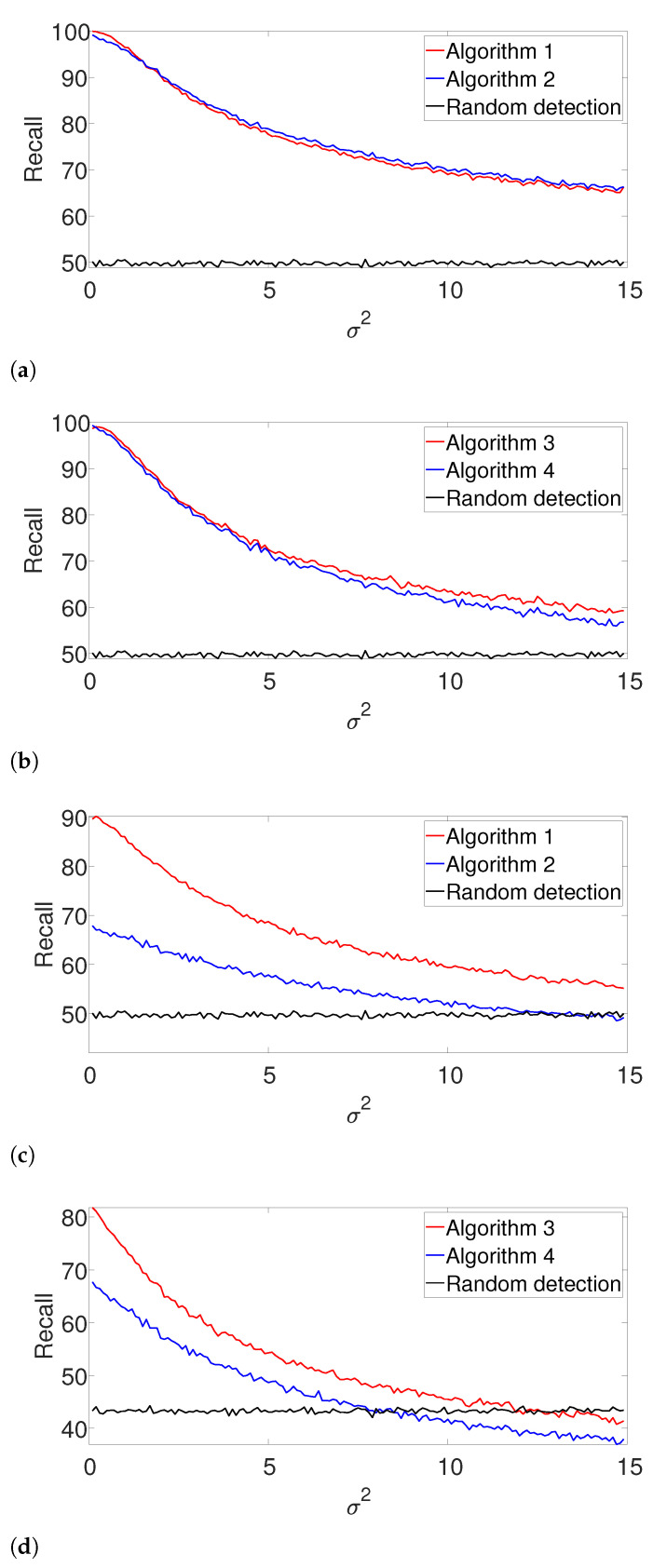
Recall for the arbitrarily spaced model where the signal length is unknown. Here, $\hat{L}$ denotes the assumed length of the signal. (**a**) $\hat{L}/L=0.8$, known *K*. (**b**) $\hat{L}/L=0.8$, unknown *K*. (**c**) $\hat{L}/L=1.3$, known *K*. (**d**) $\hat{L}/L=1.3$, unknown *K*.

**Figure A4:**
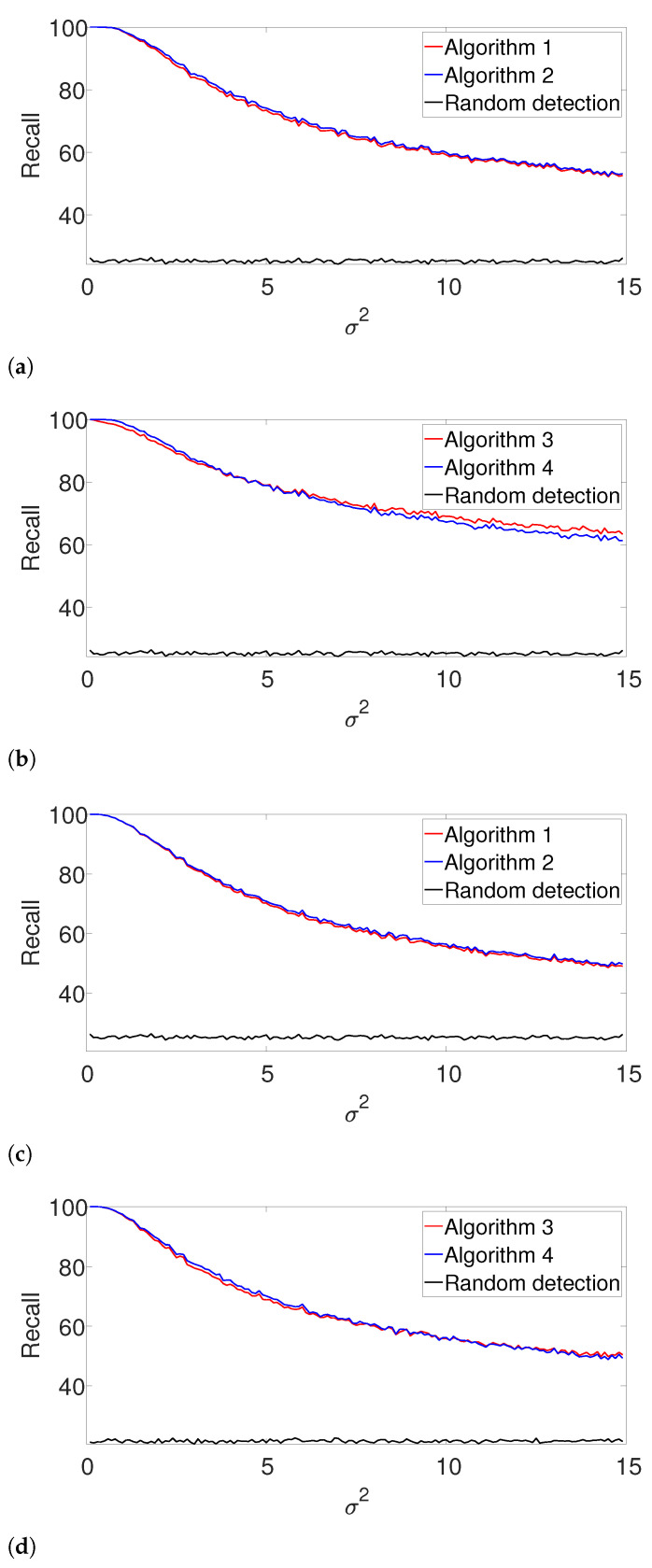
Recall for the well-separated model where the signal length is unknown. (**a**) $\hat{L}/L=0.8$, known *K*. (**b**) $\hat{L}/L=0.8$, unknown *K*. (**c**) $\hat{L}/L=1.3$, known *K*. (**d**) $\hat{L}/L=1.3$, unknown *K*.

**Figure A5:**
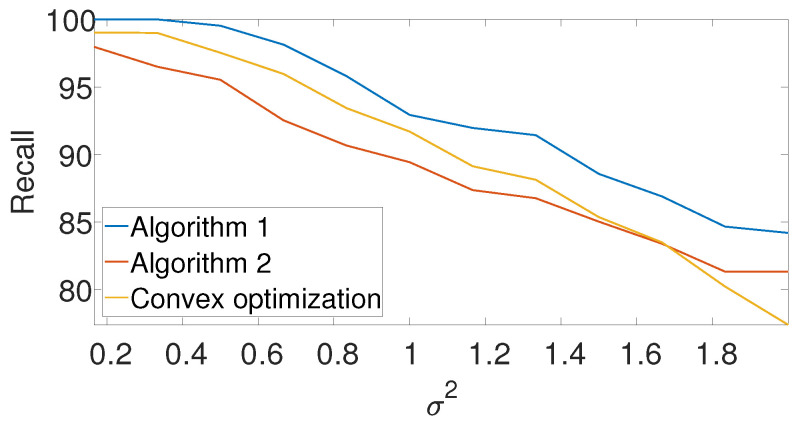
Recall for the arbitrarily spaced model as a function of the noise level for Algorithm 1, Algorithm 2, and the convex program (9).
